# Time spent on health related activities associated with chronic illness: a scoping literature review

**DOI:** 10.1186/1471-2458-12-1044

**Published:** 2012-12-03

**Authors:** Tanisha Jowsey, Laurann Yen, Paul Mathews W

**Affiliations:** 1 Australian Primary Health Care Research Institute, Australian National University, Ian Potter House, Corner of Gordon and Marcus Clarke Streets, Acton 0200, ACT, Australia

**Keywords:** Time, Time use, Health related, Chronic illness, Carer, Patient, Survey, Literature review

## Abstract

**Background:**

The management of health care, particularly for people with chronic conditions, combines the activities of health professionals, patients, informal carers and social networks that support them. Understanding the non-professional roles in health management requires information about the health related activities (HRA) that are undertaken by patients and informal carers. This understanding allows management planning that incorporates the capacity of patients and informal carers, as well as identifying the particular skills, knowledge and technical support that are necessary. This review was undertaken to identify how much time people with chronic illness and their informal carers spend on HRA.

**Methods:**

Literature searches of three electronic databases (CINAHL, Medline, and PubMed) and two journals (Time and Society, Sociology of Health and Illness) were carried out in 2011 using the following search terms (and derivatives): chronic illness AND time AND consumer OR carer. The search was aimed at finding studies of time spent on HRA. A scoping literature review method was utilised.

**Results:**

Twenty-two peer reviewed articles published between 1990 and 2010 were included for review. The review identified limited but specific studies about time use by people with a chronic illness and/or their carers. While illness work was seen as demanding, few studies combined inquiry about both defined tasks and defined time use. It also identified methodological issues such as consistency of definition and data collection methods, which remain unresolved.

**Conclusions:**

While HRA are seen as demanding by people doing them, few studies have measured actual time taken to carry out a comprehensive range of HRA. The results of this review suggest that both patients with chronic illness and informal carers may be spending 2 hours a day or more on HRA. Illnesses such as diabetes may be associated with higher time use. More empirical research is needed to understand the time demands of self-management, particularly for those affected by chronic illness.

## Background

The management of health care for people with chronic illness is a time consuming business for both patients and carers. It is usually carried out in the home, or from the home, and is largely invisible to institutional health care providers. The Serious and Continuing Illness Policy and Practice Study (SCIPPS) undertook qualitative research with people living with chronic illness to understand their experiences and interactions with the Australian health system [1]. The study sample consisted of people diagnosed with, or caring for someone with type 2 diabetes mellitus, chronic heart failure (CHF) and/or chronic obstructive pulmonary disease (COPD). One of the findings that emerged was that both patients and informal carers described experiencing a significant time burden due to managing chronic illness. They reported a constant sense of having to juggle the commitments in their lives, and saw the demands of managing health related activities (HRA) as a key element in that struggle.

The idea of ‘illness work’ carried out by people with chronic illness has been a key concept in the literature since Corbin and Strauss' [2] foundational qualitative study. The three types of ‘illness work’ they identified include 1) “regimen work, crisis prevention and management, symptom management, and diagnostic-related work”; 2) everyday life work, that includes practical tasks “that keep the household going”; and 3) biographical tasks that are done as the person and their family re-conceptualise and re-construct the ‘story’ about their lives.

This concept identifies work domains of people affected by chronic illness, but does not identify specific HRA undertaken, or how much time people spend on doing them. Information about the time demands of health management have implications for many life areas, such as patient or carer capacity to stay in the workforce, to manage family and social activities, or to maintain usual domestic and personal activities. Determining their time use may assist health providers to coordinate and manage formal care in a way that optimises time use for both health care providers and health care receivers.

Information concerning health and about time use is sought through national surveys of many countries, as can be seen from the data base of time use surveys held by the Multinational Time Use Survey at the Centre for Time Use Research in the UK [3]. However, the health data tend to be reported in aggregate with other activities, or are general rather than specific.

The Australian Bureau of Statistics (ABS) runs a number of national surveys that include questions on health, caring and time use. The Australian Time Use Survey [4] asks respondents to complete diaries about time, and includes as health activity personal medical care (taking medications, injections, vitamins, exercising for specific conditions, reading or writing in relation to personal medical care, preparing medications), rest because of illness, and health treatments (not mainstream conventional medicine with the exception of ante-natal classes). Health care, in the ABS Disability, Ageing and Carers Survey, includes foot care, and “other tasks, such as: taking medication or administering injections; dressing wounds; using medical machinery and manipulating muscles or limbs”. Statistics Canada in their General Social Survey - Time Use utilise a diary to record personal medical care (by self, other person in the home, or formal care), but do not provide a detailed definition of the specific activities included. The UK Household Survey similarly uses a diary to identify activity undertaken at multiple points in time, but does not specify on its public website how activities are coded.

This review aims to summarize the current literature that:

1. specifies HRA undertaken by people with chronic illness (patients hereafter) and their informal carers; and

2. quantifies the time spent or required to carry out HRA.

## Methods

A scoping literature review was undertaken during January to May 2011. Scoping reviews are useful studies that summarise what is known on a specific topic and are often followed by systematic literature reviews [5]. In this scoping review literature has been collected, evaluated and presented according to methods laid out for rapid review by Arksey and O’Malley [4]. Both qualitative and quantitative studies have been included for review.

We conducted an electronic literature search of peer-reviewed English-language articles in the *Medline*, *CINAHL* and *PubMed* databases which contained the desired terms in the title, abstract or key words. Furthermore, two journals *Sociology of Health and Illness* and *Time and Society* were hand-searched in order to locate relevant articles not catalogued in the databases.

The following search terms, and derivatives, were used: Chronic AND time AND treatment/management AND consumer/patient/carer AND health. The full set of terms and derivatives are shown in Table 1. We decided to use the terms “chronic illness” and “chronic disease” rather than “long term conditions” to provide a specific focus on HRA linked to chronic illness. In addition, we included the specific illnesses of diabetes, heart disease and chronic obstructive pulmonary disease as they were sentinel diseases in our main study.

**Table 1:** Search strategy

**Search terms:**		
Chronic disease OR chronic illness OR diabetes OR chronic heart failure OR chronic obstructive pulmonary disease		
Time use OR time management OR waiting time OR time burden AND		
Health treatment OR health consultation OR management OR self-manag* AND		
Health care consumer OR patient or carer AND		
Health OR health care OR primary health care OR access		
**Inclusion criteria**	**Exclusion criteria**	**Resources searched**
· English language	· Concerned with health professional time only	Medline
· Peer reviewed	· Non-specific descriptions of time and health related activities	PubMed
· Publication dates between 1990 and 2010		CINAHL
· Concerned health related activities undertaken by the individual with chronic illness and/or a carer		Two journals:
· Reported qualitative or quantitative findings		· Time and Society;
		· Sociology of Health and Illness
		Snowballing based on references in selected articles and “related articles”

**Figure 1:**
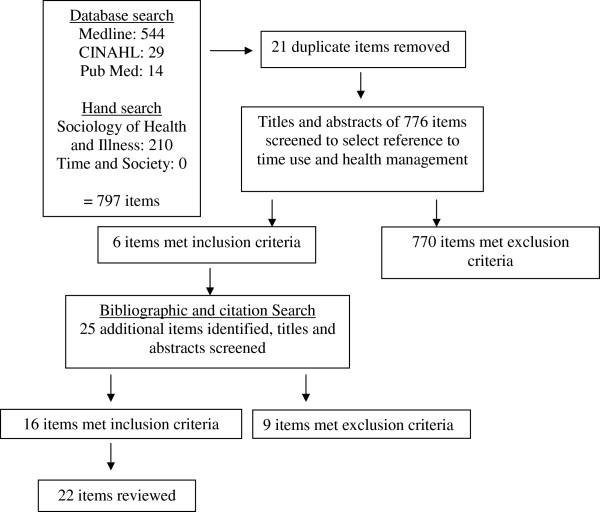
**Time costs of health related activities associated with chronic illness: a literature review.** Article selection process.

As illustrated in Figure 1, searches identified 29 items in *CINAHL*, 544 items in *Medline*, 14 items in *PubMed*, 210 items in *Sociology of Health* &*Illness*, and zero items in *Time* &*Society*. Twenty-one duplicated items were identified, leaving 776 unique references.

Two stages of screening were used to identify those studies that matched the inclusion and exclusion criteria shown in Figure 1. Using and extending the ABS definition of personal medical care, we looked for activity that would meet that definition, in addition to activity related to contact with non-inpatient health services. HRA were included if they were carried out by an individual with a chronic illness or a carer; and, as stated above, were concerned with personal health care including monitoring, management/treatment; or directed to activities undertaken to support health, including travel to and attendance at health services. Articles were excluded if they dealt with health professional, rather than health service user time, or where no specific activities or times were included.

From the 776 articles identified in the database and journal searches, only 6 articles met the inclusion criteria. Of those excluded, almost all addressed either HRA or time, but without providing both specific activity and specific time. One article addressed health professional time, rather than patient/carer time. A further 25 articles were identified by bibliography and citation-searches of the 6 included articles, from which a further 16 articles met the inclusion criteria. We selected a final set of 22 articles for full review.

All authors were engaged at each stage of the design and conduct of the review. Each search was run by all reviewers to ensure consistency and certainty of data extraction since fewer articles were identified than we expected. Articles which met the inclusion criteria were read by all three reviewers before being included for review. Articles included for review were analysed for emerging themes. We manually extracted details of the time use measures.

Having read all 22 papers in detail, we identified three principle themes: 1) time spent by individuals on specific HRA; 2) time spent by carers on specific HRA; and 3) the methodological difficulties associated with time use studies.

Where studies reported time spent in hours, such as ‘1.43 hours’ we have taken this to mean one hour and 43 minutes rather than one hour plus 0.43 of an hour. We have used the same convention when the time is reported in minutes, so that 19.02 minutes means 19 minutes and 2 seconds.

## Results

### Study characteristics

Table 2 and Table 3 detail the scope of reviewed articles.

**Table 2:** Scope of articles addressing time use and chronic disease

	**N(n=22)**	**%**
**Region**		
United States	12	48
Australia	7	28
United Kingdom	1	4
Canada	1	4
Italy	1	4
**Time users**		
Patient only	12	48
Informal carer only	5	20
Patient and informal carer	3	12
Other (method focus)	2	8
**Article type**^		
Method used: survey	13	52
Method used: time use diary	3	12
Method used: qualitative (interview or focus group)	7	28
Method used: descriptive	2	8
Method used: modeling or RCT	2	8
Methodological issues (Informal carer)	4	16

^ Some articles used more than one method.

**Table 3:** **Brief description of articles included for review** (**in alphabetical order**)

	**Author, Title & Year**	**Country**	**Disease (if specified)**	**Design and methods- survey, RCT, qualitative study**	**Sample size**
1	Bittman & Thomson. Invisible Support: The determinants of time spent in informal care. 2000. [11]	Australia		Method: survey. Quantitative analysis of ABS Time Use Surveys and Survey of Disability, Ageing and Carers. Uses this secondary data re: time burden/ use among carers, with a major focus on non-coresidential vs coresidential care.	14,315 carers
				Characteristics of care recipients and informal carers include: living arrangements of carers and care recipients, level of disability, household income, poverty rates and effects on various lifestyle features.	
2	Bittman et al. Making the invisible visible. The life and time(s) of informal caregivers. 2004. [12]	Australia		Method: survey and diary. Quantitative data from surveys and diaries from Canadian (N= 10,749) and Australian (N= 14,000 approximately) bureaux used to explore and compare time burden and time use among carers and non-carers, as well as methodological issues in obtaining data and measuring time use and caring activities.	Multiple samples: patients and carers
				Main variables are co-residency and non-care responsibilities.	
3	Bittman, M. et al. The time cost of care. 2005. [13]	Australia		Method: survey and diary. This paper contrasts two different measures of care time using survey questions or a diary.	Multiple samples: carers
4	Braithwaite, V. Bound to Care. 1990. [6]	Australia		Method: qualitative, descriptive and survey. Overall, takes a sociological view of what a caregiver is/does and means, it's not just tasks and burden, but a relationship and a responsibility.	138 carers
				Although dated, and focused on care-givers, does provide some early basic data on time and other burdens in caring.	
5	Corbin, J. & Strauss, A. Managing chronic illness at home: Three lines of work. 1985. [2]	USA	Mainly cardiovascular diseases, cancer, stroke, & spinal injuries.	Method: qualitative. Interviews and (auto) biographies of people with CI and their spouses.	60 couples: patients and carers
				Uses the concept of "work" in managing CIs and types of work: illness, everyday and biographical work.	
6	Ettner, S. et al. Investing time in health: do socio- economically disadvantaged patients spend more or less extra time on diabetes self-care? 2009. [26]	USA	Diabetes	Method: survey. Comprehensive survey and statistical analysis, using several variables (education, marital status, income, minority group/ethnicity status, work status, clinical characteristics) but limited to one CI; looks at only foot care, exercise and (conflates) shopping/cooking.	11,927 patients
				Objective: To examine associations between socioeconomic position and extra time patients spend on foot care, shopping/cooking, and exercise due to diabetes.	
7	Hu, P. & Reuben, D. Effects of managed care on the length of time that elderly patients spend with physicians during ambulatory visits. 2002. [24]	USA		Method: survey. Cross-sectional analysis of the 1998 National Ambulatory Medical Care Survey.	4,964 elderly patients
8	Infante, et al. How people with chronic illnesses view their care in general practice: a qualitative study. 2004.	Australia		Method: qualitative. 12 focus groups.	76 patients
				Objectives: To explore the perceptions of patients with chronic conditions about the nature and quality of their care in general practice.	
9	Ironmonger, D. The value of care and nurture provided by household work. 1994. [7]	Australia		Method: survey. Comparative statistical analysis of mainly ABS survey data of aggregates hours for aged care and related household activities	Multiple samples: carers
10	Jenkins, C. Women, work, and care giving: How do these roles affect women's well-being? 1997. [8]	USA		Method: survey. Statistical analysis of data from the 1988 National Survey of Families & Households (USA); how much time in care- giving and other activities, and effects on stress levels.	14,500 female carers
11	Langa, K., et al. Informal caregiving for chronic lung disease among older Americans. 2002. [9]	USA	Lung disease	Method: survey. Multivariable regression models using data from the 1993 Asset and Health Dynamics Study by survey.	National population-based sample of 7,443 community- dwelling elderly patients >70.
				Measurements: Weekly hours of informal care giving, and imputed cost of caregiver time.	
				The average number of hours per week of informal care was calculated for: activities of daily living (ADL); and instrumental activities of daily living (IADL).	
12	McCoy, L. Time, self and the medication day: a closer look at the everyday work of 'adherence'. 2009. [17]	Canada	HIV	Method: qualitative. 21 interviews and 16 focus-groups with people taking antiretroviral drugs.	79 patients
13	McKenna, K. et al. Comparison of time use, role participation and life satisfaction of older people after stroke with a sample without stroke. 2009. [21]	Australia	Stroke	Method: qualitative & time use diary. Interviews with 23 participants and data compared with a prior study. Interviews prompted participant recall using calendars and diaries.	23 patients >65 yrs old 1– 3 yrs post-stroke (mean age
					74.2 years, 69.6% men)
14	Paoletti, I. A half life: Women caregivers of older disabled relatives. 1999. [10]	Italy		Method: qualitative. Interviews and discourse analysis.	50 female paid and unpaid carers.
15	Pritchard, P. Doctors, patients and time. 1992. [25]	UK		Method: Descriptive. A narrative description about time and time use, different kinds of time, from both patients' and Doctors' perspective, their perceptions of the other's perceptions of time and its use and value.	N/A
16	Reed, et al. Economic evaluation of home blood pressure monitoring with or without telephonic behavioral self-management in patients with hypertension. 2010. [31]	USA	Hypertension	Method: RCT (other). A prospective economic evaluation alongside a randomized controlled trial of 636 patients with hypertension participating in the study's 3 interventions. Medical costs were estimated using electronic data representing medical services delivered within the health system. Intervention-related costs were derived using information collected during the trial, administrative records, and published unit costs.	636 patients.
17	Russell, L. et al. Time requirements for diabetes self-management: Too much for many? 2005. [18]	USA	Diabetes	Method: qualitative. A convenience sample of 8 certified diabetes educators to derive consensus-based estimates of the time required for all self-care tasks recommended by the American Diabetes Association.	8 certified diabetes educators
					(concerning patient time use)
18	Russell, L. et al. Health- related activities in the American Time Use Survey. 2007. [20]	USA		Method: survey. Compilation and statistical analysis of ATUS survey data on Health-Related Activities in America.	34,000 patients
19	Russell, L. et al. How much time do patients spend on outpatient visits?: The American Time Use Survey. 2008. [22]	USA		Method: survey. Compilation and statistical analysis of ATUS survey data on outpatient visits.	1,621 random sample of patients from 2003–06 ATUS data, age >15
20	Safford, M. et al. How much time do patients with diabetes spend on self-care? 2005. [19]	USA	Diabetes	Method: survey. Cross-sectional survey of 1482 diabetic patients enrolled in 3 northeastern United States managed care plans. Statistical analysis using and linear regressions.	1,482 diabetic patients (57.9% >55 yrs)
21	Wolf, D. Valuing informal elder care. 2004. [14]	USA		Method: modelling. Addresses through modelling the problem of attaching a monetary value to informal elder care, and why we should; uses NLTCS data to illustrate.	N/A
22	Yabroff, K. et al. Estimating patient time costs associated with colorectal cancer care. 2005. [23]	USA	Colorectal cancer	Method: survey. Quantitative, longitudinal and comparative time data for cancer/non-cancer care based on past studies and medical records (SEER-Medicare database), and estimates a monetary value of patients' time based on BLS wage rates.	75,470 patients with matched controls

Study characteristics are outlined in Table 3. Studies were conducted in five countries, with almost half the articles reporting studies from the United States of America (n=12). Twelve studies provided information about time use or time management among patients (n=12). Six studies reported carer time use only [6-11] and two articles concerned time use of both patients and carers [2,12]. Most studies reported either survey data (n=13) or qualitative data (n=7). Four articles focused on methodological issues associated with measuring time use by carers [11-14], but none focused on methodological issues associated with measuring time use exclusively by patients.

### Time spent by individuals on specific health related activities

Two studies provided information about how much time people spend on certain private or household tasks such as sleeping, leisure, grooming, and on HRA including exercise, which was reported as a separate item [15,16].

Three articles reported time use in terms of patient compliance and adherence. McCoy notes a broad range of potential reasons for noncompliance with self-care and medication regimens [17]. They conclude that medication adherence by people with chronic illness is complex and labour-intensive [17]. Russell et al. conclude that factors other than knowledge are needed to achieve necessary behavioural change and compliance. However, “scant attention has been paid to time requirements and little is known about how much time current recommendations take” [18]: 53, see also, [19].

The most comprehensive information about actual time spent on HRA was found in studies based on the USA Bureau of Labour Statistics’ American Time Use Surveys (2003–09). These surveys provide a comprehensive set of statistical data (http://www.bls.gov/tus/). Russell [20] reports that for the 11.3% of adult Americans surveyed who indicated that they had spent time on HRA in the previous 24 hour period (their ‘designated day’), the average time spent overall was 108 minutes. Those engaging in personal health self-care reported it to take an average of 86 minutes. Medical and care services reportedly took 123 minutes, and sports, exercise and recreation reportedly took 114 minutes. Those caring for others reported spending between 78 and 115 minutes in activities related to the health of others. This contrasts with the findings of McKenna and colleagues [21] in an Australian qualitative study comparing people who had suffered a stroke with those who had not that HRA was the least time consuming of their measured activities and the average time spent was around 30 minutes each day.

Two studies identified time spent attending health service appointments. Russell et al. [22] reported on three years of the American Time Use Survey data, showing that of 1621 patients seeking medical care on a ‘designated day’, mean time spent was 35 minutes for travel, 42 minutes for waiting and 74 minutes for receiving services. Accompanying carers spent an average of 124 minutes for each encounter. Yabroff et al. [23] estimated patient time costs associated with colorectal cancer care using data from several surveys and physician-reported time data. They estimated that each office visit required 1.43 hours or 1 hour 43 minutes for patients in metropolitan areas and 1 hour 58 minutes for those living outside metropolitan areas. Yabroff et al. [23] cite other instances of time measurement, associated with screening activity.

One study, Hu & Reuben [24], focused on the length of time elderly patients spent with physicians during ambulatory visits and reported an average of 19.02 minutes for elderly patients, 27 minutes for new patients and 18.03 minutes for established patients, concluding that the effects of managed care on the duration of visits appear to be related to the structure of the managed care plan. Pritchard [25] also focused on consultation times and how patients and GPs negotiated and managed this time use, but did not specify the time actually spent.

Two studies examined the additional time spent on HRA due to diabetes, over and above the time people would usually spend on HRA. Using surveys and phone interviews, Ettner et al. [26] studied the impact of socioeconomic status on time spent on self care for people with diabetes, looking specifically at time spent on foot care, shopping for and cooking special foods, and undertaking recommended exercise. Ettner et al. found that those spending "extra time" on HRA as a result of having diabetes spent on average an extra 13.41 minutes daily on foot care, 38.57 minutes on exercise and 42.42 minutes on shopping and cooking. About two thirds of Ettner et al’s respondents spent extra time on foot care and exercise, and about half spent time on shopping and preparing food specifically for their health condition. Safford et al. [19] also used surveys to identify HRA of patients with diabetes. They report similar findings to Ettner et al., with 75% of patients spending at least 19 minutes daily on self-management. The focus of their discussion is on how many patients *did not* spend time on specific recommended activities.

Only two studies estimated the *overall* time required for HRA over a 24 hour period. Russell et al. [18] used a convenience group to establish that the time required for self care of diabetes was approximately 120 minutes daily. Safford [19] quantified how much time diabetics spent on self care, with a mean time of 58 minutes per day. These included foot care (13 minutes), exercise (32 minutes) and food shopping and preparation (48 minutes). Safford also identified that over a third of respondents spent no time on either foot care or exercise, and over half spent no time on food shopping and preparation.

In summary, studies included for review suggest that over a 24 hour period patients are likely to spend 86 minutes on HRA [20]; less time if they have had a stroke [21], more time if they have diabetes [18,19,26]. If patients also engage in exercise they spend in the order of 35 minutes each day. Those who care for someone else spend an extra 78 to 115 minutes daily [20]. Access to health services is not a daily occurrence for most, but each event may require between 104 minutes and 151 minutes, which includes 35 minutes for travel to health services, 42 minutes for waiting for health services and 74 minutes receiving health services [22]. If the patient lives in a metropolitan area Yabroff suggests the time to access health services is 103 minutes [23]. So, if a patient with diabetes engages in HRA (including exercise), and also accesses health services on a given day they may spend (120 diabetes self-care + 35 exercise + 151 access) 306 minutes (5.06 hours) doing so. If they also care for someone else on that day their care duties could consume another 78–114 minutes, a total of almost 7 hours.

Combined, these studies present a picture of high time expenditure on daily HRA for those with diabetes. Consistent definition is lacking about the specific tasks carried out by people in managing their health, as is the time taken, every day, or over longer periods, to do them. In addition, apart from estimates of time needed for care of diabetes, there is almost no information available about the time costs of health management for people with other chronic conditions, and no information concerning time use of people with multiple conditions.

### Time spent by carers on health related activities

A third of the articles included for review approached time use specifically from a *carer*’*s* perspective [6-10] or in combination with patients [2,20]. A further two papers [27,28] provided characteristics or profiles of carers, but did not report their time use. None of these articles specified the HRA carried out or provide specific measures of carer time spent on HRA *for self*. Only one paper reported carer time spent on HRA for care recipient [20]. Russell et al. [20] used ATUS data to measure time use among American adults, and found that people reported spending 78–115 minutes per day on HRA (unspecified) in support of both household and non-household members.

Bittman & Thomson [11] and Bittman et al. [12,13] found the ABS’ *Disability*, *Ageing and Carers Survey* (2003) and *Time Use Survey* (1998) contained limited and problematic information about the time devoted to care. The broad ABS categories include meal preparation, property maintenance, housework, transport, paperwork, health care, cognition or emotion, communication, and mobility, but the data provide no details of what some of these may involve, nor how much time is spent for each except with cross tabulations with other variables such as carer’s and care recipient’s age group, disability level (which may or may not include a chronic illness), or years of care provided, at best revealing an average weekly range of 6 to 27 hours, climbing to over 105 hours depending on the severity of disability. “Consequently, there is hardly any systematic knowledge about what determines the quantity of labour required for informal care, its nature or its intensity and the demands it places on families. Therefore it is not possible to estimate the demands placed on carers, how they vary according to changes in circumstances and to make informed judgements about the supply of caring labour” [13]: 57.

Langa et al. [9] report that individuals with chronic lung disease and activity limitations received an additional 5.1 h/wk of informal care compared to those with no lung disease, and therefore, if the full societal costs of chronic lung disease are to be calculated then the costs to families and society must be accounted for.

## Discussion

### Comparison - limitations of studies included for review

Studies included in this review reported encountering multiple methodological difficulties that limited their capacity to comprehensively measure total time use for patients or carers. Key limitations of the studies included in this review concern secondary analysis, mode of time measurement, values attributed to time, and the lack of consistency in what is measured across studies.

Studies that undertook secondary analysis of large datasets were unable to report detailed information on the elements of HRA because they were not included in the survey questions. As an example, the Australian National Health Survey (2007–2008) records activities undertaken by people with self-reported conditions, but does not identify what time is taken to carry out any of these activities [29]. Similarly, the Australian Time Use Survey: How Australians Use Their Time, 2006 [30] identifies health as one of the activities on which time is spent, but does not specify or quantify particular activities or the time taken (see for example, Bittman et al., 2005).

Studies were also limited by their mode of time measurement. Bittman et al. [13] examined two data sets on time use from the Australian Bureau of Statistics that utilized two different methods (the 1998 *Australian Survey of Disability*, *Ageing and Carers*, which included time use estimates based on a question concerning hours spent weekly on activities, and diary estimates from the 1997 national *Australian Time Use Survey*). They argue there is an inconsistency in the two estimates, and this may be because of methodological reasons such as; "the time use data may well miss out some supervisory time, and not always indicate the extent to which carers rearrange their schedules to be nearby to the care recipient in case they are needed" [13]: 62. Regardless of which mode of time measurement is utilised by a study there are likely to be aspects of time use which, for whatever reason, are not captured. With regards to the studies included in this review, this limitation does not reflect the quality of research undertaken as much as the complex nature of defining and measuring time use.

The reviewed studies presented different classifications of time use. Underestimation of patient time costs may result from misclassification. For example, Bittman et al. [12] report that food preparation and cooking may be under-reported or classified as a “domestic” activity rather than a caring task. In other cases there may be incomplete information on travel or service time, as well as counting multiple therapeutic claims or procedures within a short period as one episode; and in monetary terms, the extrapolation of the wage rates used in the computation of the value of patient-costs take no account of how sick or retired persons may value their time consumption. This is evident in Reed et al’s [31] paper that compared three forms of care for hypertension, valuing the patients’ time, based on information from the USA Bureau of Labor Statistics. They showed that the three interventions were cost-additive to the health-care system; that patients’ time costs were not trivial, and the interventions took no account of how time was valued by patients.

Extending on this problem of classification, subjective terminology was used in some time use surveys. Jenkins [8] identifies several limitations of question styles employed in the 1988 National Survey of Families and Households, such as the restrictions created by respondents being asked if they care for someone who is seriously ill or disabled. 'Seriously' is a somewhat subjective term, and all data concerning care recipients which was not deemed 'seriously ill' by respondents was therefore not measured. McKenna et al. [21] in their study of time use after stroke excluded people from participating if they had cognitive impairment; however they note that as cognitive impairment is a result of stroke in 60% of cases this somewhat limited the generalisability of their data. Similarly, Bittman and Thompson [11] note that the Australian Bureau of Statistics data does not separate caring for disabled/handicapped from caring for chronically ill people, limiting the specificity of Bittman and Thompson’s time use analysis.

Other methodological limitations noted in the literature were concerned with whether or not studies were longitudinal or cross-sectional [23], or if they compared time use amongst those with and without chronic illness [32]. Schoefield et al. [28] note that time use studies are often based on small samples, reducing the power of the findings. Folbre (2006) argues that small time use studies may be gender or otherwise biased.

### Discussion of findings

This review set out to establish, from existing literature, which HRA undertaken by patients and informal carers has been measured; and how much time they are reported to have spent on HRA. The kinds of HRA that are measured and reported in the reviewed studies have limited alignment with the ‘illness work’ outlined by Corbin and Strauss [2]. Some studies did measure exercise and access to health services (for example, 20), which could be seen as part of the first kind of ‘illness work’, which is regimen and diagnostic related activity. Others looked broadly at HRA, which is the everyday life work described as the second kind of ‘illness work’; however in these papers time spent on specific HRA was seldom reported. Diabetes care work was measured by two studies and others included medication adherence (for example, 17, 26). Other types of HRA such as food preparation and consumption or obtaining medicine prescriptions were not reported specifically. The biographical tasks outlined in the third kind of ‘illness work’ did not have a strong presence in the studies, although McCoy [17] and Paoletti [10] make a start. It is likely that studies focusing on the biographical tasks of ‘illness work’ do exist but did not meet our inclusion criteria. Such papers could inform researchers of specific HRA that are not currently measured.

There are only a small number of studies which reported patient and carer time use in relation to chronic illness. Five key articles [6,11,18,19,26] detail that time use and how it affects lifestyle and wellbeing. Armstrong [33] observed that there is a lack of accurate and comprehensive information about the time spent by people who themselves have a chronic illness in looking after their health. It is a point emphasized by Singleton [34]: 692 who says that “the voices of patients are disturbingly absent” from the literature on time use, and which is addressed only superficially by, for example, Corbin & Strauss [2], McCoy [17] and Paoletti [10]. This review identified a small number of articles, which, when combined, lead us to conclude that these observations by Armstrong, Singleton, and others remain the case.

These limitations notwithstanding, the available literature indicates that the time use and burden associated with managing a chronic illness is sizable [12,23,31]. Patients with chronic illness and informal carers may be spending 2 hours a day or more on HRA. Measurements of time spent on specific activities are needed to inform our understanding of the real time burden associated with ‘illness work’. Additionally, the available literature indicates that approximately 2 hours are required for every health system contact, to which can be added the same amount for the time of a carer accompanying the patient. These estimates of time spent on HRA are likely to under represent actual patient and informal carer time use, and as Yabroff suggests, may increase with progression of the illness. If time were consumed in one block it might be more readily accommodated into a person's life style. However, HRA are often spread across a whole day and therefore may be found to be simply disruptive, a burden, and de-motivating, as McCoy [17] has demonstrated for HIV-infected persons. Additionally, we know almost nothing of how much illness-related time burden impacts people’s overall wellbeing, motivation, and even access to medical care.

## Conclusion

This review shows that little is reported about the specific activities undertaken by patients and carers to manage chronic illness. The results suggest that patients with chronic illness and informal carers may be spending 2 hours a day or more on HRA. For specific chronic illnesses, such as diabetes, for which some estimates of time use exist, time use may be higher. More precise and rigorous measurement of activities and commensurate time commitments with which carers and the chronically ill engage are necessary to better understand the work of chronic illness, its impact on life choices, and its true cost.

## Competing interests

The authors declare no competing interests. The funding organisation (NHMRC) had no role in the study design, data collection, analysis and interpretation, or the writing and publication of this article.

## Authors’ contributions

TJ made substantial contributions to conception and design, acquisition of data, primary analysis and interpretation of data; and was heavily involved in drafting the manuscript and revising it critically for important intellectual content. LY conceived of the study; made substantial contributions to conception and design; undertook primary analysis and interpretation of data; and was heavily involved in revising the manuscript critically for important intellectual content. PM contributed to acquisition of data, analysis and interpretation of data; and was involved in initial drafting stages of the manuscript. All authors read and approved the final version of the manuscript.

## Authors information

This research was undertaken by three members of the Serious and Continuing Illness Policy and Practice Study at the Australian National University. The authors have training in anthropology, psychology and health services research. TJ is undertaking postdoctoral research concerning experiences of time and chronic illness at the Australian National University and this paper forms part of her research.

## Pre-publication history

The pre-publication history for this paper can be accessed here:

http://www.biomedcentral.com/1471-2458/12/1044/prepub
